# Quercetin abrogates chemoresistance in melanoma cells by modulating ΔNp73

**DOI:** 10.1186/1471-2407-10-282

**Published:** 2010-06-11

**Authors:** Thilakavathy Thangasamy, Sivanandane Sittadjody, Geoffrey C Mitchell, Erin E Mendoza, Vijayababu M Radhakrishnan, Kirsten H Limesand, Randy Burd

**Affiliations:** 1 Department of Nutritional Sciences, University of Arizona, Tucson, AZ 85721, USA; 2 Cancer Biology Interdisciplinary Program, University of Arizona, Tucson, AZ 85721, USA; 3 Arizona Cancer Center, University of Arizona, Tucson, AZ 85721, USA

## Abstract

**Background:**

The alkylating agent Dacarbazine (DTIC) has been used in the treatment of melanoma for decades, but when used as a monotherapy for cancer only moderate response rates are achieved. Recently, the clinical use of Temozolomide (TMZ) has become the more commonly used analog of DTIC-related oral agents because of its greater bioavailability and ability to cross the blood brain barrier. The response rates achieved by TMZ are also unsatisfactory, so there is great interest in identifying compounds that could be used in combination therapy. We have previously demonstrated that the bioflavonoid quercetin (Qct) promoted a p53-mediated response and sensitized melanoma to DTIC. Here we demonstrate that Qct also sensitizes cells to TMZ and propose a mechanism that involves the modulation of a truncated p53 family member, ΔNp73.

**Methods:**

DB-1 melanoma (p53 wildtype), and SK Mel 28 (p53 mutant) cell lines were treated with TMZ (400 μM) for 48 hrs followed by Qct (75 μM) for 24 hrs. Cell death was determined by Annexin V-FITC staining and immunocytochemical analysis was carried out to determine protein translocation.

**Results:**

After treatment with TMZ, DB-1 cells demonstrated increased phosphorylation of Ataxia telangiectasia mutated (ATM) and p53. However, the cells were resistant to TMZ-induced apoptosis and the resistance was associated with an increase in nuclear localization of ΔNp73. Qct treatment in combination with TMZ abolished drug insensitivity and caused a more than additive induction of apoptosis compared to either treatment alone. Treatment with Qct, caused redistribution of ΔNp73 into the cytoplasm and nucleus, which has been associated with increased p53 transcriptional activity. Knockdown of ΔNp73 restored PARP cleavage in the TMZ treated cells, confirming its anti-apoptotic role. The response to treatment was predominantly p53 mediated as the p53 mutant SK Mel 28 cells showed no significant enhancement of apoptosis.

**Conclusion:**

This study demonstrates that Qct can sensitize cells to TMZ and that the mechanisms of sensitization involve modulation of p53 family members.

## Background

Melanoma has been categorized as the most aggressive form of skin cancer [1] and its incidence has increased worldwide over the last 50 years. In the United States, this form of cancer is the fifth and sixth most common cancer in men and women, respectively, and has an estimated average lifetime risk of 1 in 75 [2,3]. Dacarbazine (DTIC) is considered one of the most effective chemotherapies for metastatic melanoma, with response rates ranging between 10-20%; however, lower response rates (7-8%) and a 6-year survival rate of 2% have been reported [4]. Unfortunately, the poor response of melanoma to chemotherapy is also accompanied by systemic toxicities that lead to poor quality of life for patients.

Temozolomide (TMZ), a DTIC derivative, is a second-generation imidazotetrazine alkylating agent that is hydrolyzed to the active metabolite 5-(3,3-methyltriazen-1-yl) imidazole-4-carboxamide which further decomposes into a DNA methylating species [5]. TMZ represents a new analogue of DTIC with more desirable properties because it can enter the cerebrospinal fluid and does not require hepatic metabolism for activation. It has the same cytotoxic activity as DTIC, which results from its ability to add a methyl group to the O^6 ^position of guanine in genomic DNA [6]. TMZ has been approved for the treatment of brain metastasis and has demonstrated clinical activity against melanoma, but overall it yields response rates similar to that of DTIC.

To improve response rates without increasing toxicities, various biological therapies have been considered for use in combination with this class of chemotherapy. Polyphenol compounds are of particular interest in combination therapies because they can be readily activated by oxidases overexpressed in many tumors [7,8]. Quercetin, for example, is a naturally occurring polyphenol that becomes activated in tyrosinase expressing cells such as melanoma. Qct is an established anticancer compound that exhibits anti-proliferative properties in numerous cancer cell lines [9] and animal models [10]. Enzymatic activation of Qct by tyrosinase specifically enhances its anti-tumor activity in melanoma cells [11] and increases the effectiveness of additional cytotoxic compounds [12].

The chemosensitizing effect of Qct has yet to be utilized clinically, but its use as an adjuvant to conventional chemotherapy could potentially enhance the therapeutic ratio in melanoma cells by increasing tumor cell kill in tyrosinase expressing cells while having little effect on normal tissue toxicity. The mechanism of tumor cell kill by chemotherapeutic drugs is in part through the induction of apoptosis [13]. Apoptosis is largely mediated by the tumor suppressor gene p53, and numerous cancer cell models indicate that chemosensitivity is positively correlated with the induction of p53. In most melanoma cells the p53 gene is wildtype, which further supports the use of apoptosis inducing agents in the treatment of melanoma.

In melanoma, p53 protein levels increase with tumorigenesis and development [14], and despite the presence of functional p53, melanoma is generally regarded as a chemoresistant tumor type. One possible explanation for the development of the resistant phenotype could be through the upregulation of p53 antagonists, such as truncated p53 family members [14]. The p73 protein is a homolog of p53, and has antitumor effects in various cancerous cells, which are mediated through cell cycle arrest and the induction of pro-apoptotic target genes [15]. However, several isoforms of p73 exist, including a truncated form that act as a p53 antagonist. The N terminal truncated form (ΔNp73) acts as an antagonist to p53 by localizing to the nucleus and preventing transcription of p53-responsive genes, such as Bax [16]. Here, we demonstrate that ΔNp73 is induced by TMZ and prevents p53-mediated apoptosis and cell death. Chemoresistance is reversed by Qct, and we therefore propose a mechanism by which Qct abrogates the inhibitory effects of ΔNp73 by modulating the protein and altering its localization.

## Methods

DB-1 melanoma cells were developed from lymph node biopsies from metastatic patients at Thomas Jefferson University, Philadelphia [17]. The cells were grown in *α*-minimum essential medium (MEM) complete medium in a 5% CO_2 _incubator at 37°C and stably express the pcDNA3 vector as previously described [12]. SK Mel 28 (mutant for p53) and SK Mel 5 (wild type for p53) melanoma cell lines were obtained from American Tissue Culture Collections (Rockville, MD, USA). Quercetin (3, 3, 4, 5, 7-pentahydroxy flavone), *α*-MEM and dimethylsulfoxide (DMSO) were purchased from Sigma, (St. Louis, MO, USA). TMZ was a kind gift from The Developmental Therapeutics Program, National Cancer Institute (Bethesda, MD, USA). Antibodies for Bax, p53 and Tyrosinase for western blotting were obtained from Santa Cruz Biotechnology (Santa Cruz, CA, USA). Antibodies for phosphorylated p53 (at ser 15, 37, 392), phosphorylated ATM (ser 1981), DNApk and PARP were obtained from Cell Signaling (Danvers, MA, USA). Antibody for GAPDH was purchased from Millipore-Chemicon (San Francisco, CA, USA). p73 antibody for western blotting and immunocytochemistry (ICC) was obtained from IMGENEX (San Diego, CA, USA). Sterile DMSO (0.1%) dissolved in *α*-MEM complete medium was used as vehicle. Quercetin and TMZ were prepared in sterile filtered DMSO.

### TMZ and Qct treatment

TMZ (20 mg/ml) was dissolved in DMSO and then dissolved in *α*-MEM complete medium and sterile filtered after adjusting the pH to 7.4. For combination treatments the cell lines were treated with TMZ 400 μM for 48 hr followed by Qct 75 μM for 24 hr.

### Western blotting

Cell lysates were electrophoresed in 7 and 10% NUPAGE gels (Invitrogen Corp., CA, USA). Separated proteins were electrophoretically transferred to Hybond PVDF membrane (Amersham Pharmacia Biotech, UK) and the membrane was blocked for 1 hr by incubating the membrane in I-block (Tropix kit, Applied Biosystems, CA, USA). Primary antibodies were used at the dilutions which the manufacturers suggested. ALP conjugated goat anti-rabbit IgG was used at a dilution of 1:10000 for antibodies for phospho-p53, DNApk, Bax and PARP whereas, anti-mouse IgG was used for Total p53, Total p73, phospho-ATM and GAPDH at a dilution of 1:10000. Western detection was carried out using CDP star from Tropix kit, Applied Biosystems, CA, USA.

### Annexin V-FITC staining

The p53 wild type and mutant cell lines were grown up to 50% confluency and were treated as mentioned above. Apoptosis was determined using Fluorescein isothiocyanate-conjugated Annexin V (Annexin V-FITC)/Propidium Iodide (PI) apoptosis detection kit (R&D systems, Minneapolis, MN, USA) as per manufacturer's instructions. Approximately 5 × 10^5 ^cells were resuspended in 100 μl of 1× binding buffer, 1 μl of Annexin V-FITC and 10 μl of propidium iodide. After 15 min incubation at room temperature in the dark, 400 μl of 1× binding buffer was added and the cells positive for Annexin V-FITC and/or PI were analyzed using a BD FACS flow cytometer.

### RNA isolation and RT-PCR

Homogenization of cells and isolation of RNA were performed using QIAshredder spin columns and an RNeasy Kit as instructed by the manufacturer (Qiagen, Valencia, CA). 1 μg of RNA was reverse transcribed using a Super Script III Kit as instructed by the manufacturer (Invitrogen, Carlsbad, CA) and diluted 1:5 for subsequent analysis. The following PCR reaction mix was used: 5 ul of diluted cDNA, 1 ul of mixed forward and reverse primers (10 uM each), 12.5 ul SYBR Green (Qiagen), and nuclease-free water to a final volume of 25 ul. For non-quantitative PCR, cDNA was amplified for thirty cycles. Forty cycles of quantitative PCR were performed (95°C for 15 seconds, 54°C for 30 seconds, 72°C for 30 seconds) using an iQ5 Real-Time PCR Detection System (BioRad, Hercules, CA) and run on a 1% agarose gel. Real-time PCRs were run in triplicate for each cDNA sample using an iQ5 Real-Time PCR Detection System. Forty cycles of PCR were performed (as described above) with fluorescence detection during the 72°C step at each cycle. The data were analyzed using the 2^-ΔΔCt ^method [18], and results were normalized to S15, which remains unchanged in response to treatment. Normalized values were plotted as relative fold over untreated. The following primers were purchased from Integrated DNA Technologies (Coralville, IA, USA): S15 [19] transcriptionally active p73 (TAp73) [20] and ΔNp73 [21].

### Immunocytochemistry

The cells were grown on cover slips in 100 mm tissue culture dishes and were treated with TMZ and Qct as mentioned above. The cover slips were placed in 6 well dishes and washed with PBS and fixed with 95% ethanol and 5% glacial acetic acid for 5 min. The slides were rinsed with PBS and were incubated with 0.5% of Triton X-100 in PBS for 10 min to permeabilize the membranes and rinsed again. After blocking the endogenous peroxidase with 3% hydrogen peroxide (H_2_O_2_) in PBS for 20 min the cover slips were processed according to staining procedure of the manufacturer's protocol for Histostain plus kits, Zymed Laboratories (Invitrogen, CA, USA). Total p73 antibody was used in the dilution of 1:250.

### siRNA transfection

siRNA transfection was carried out according to manufacturer's protocol (Invitrogen, CA, USA). Cells were grown up to 50% confluence in antibiotic free medium in 100 mm dishes. Stealth RNAi for p73 at varying concentrations (1 nM-50 nM) was diluted in 1.5 ml OPTI-MEM I reduced serum. 30 μl of Lipofectamine™ 2000 was diluted in 1.5 ml OPTI-MEM I reduced serum medium, mixed gently. After 5 min incubation at RT, diluted oligomer was combined with diluted Lipofectamine™ 2000, mixed gently and incubated at room temp for 20 min. The oligomer-Lipofectamine™ 2000 complexes were added to each plate in OPTI-MEM I reduced serum medium by mixing gently and by rocking the plate back and forth. After 6 hrs incubation in a 5% CO_2 _incubator at 37°C, the plates were subjected to TMZ treatment for 48 hrs without removing the complexes. BLOCK-iT Fluorescent oligomer was used as positive control.

### Transient transfection with Tyrosinase

DB-1 cells were transiently transfected with 6 μg of tyrosinase or pcDNA3 DNA using Lipofectamine™ 2000 in serum free OPTI-MEM medium for 5 hr followed by leaving the complex in Neomycin containing α MEM complete medium for at least 18 hrs as demonstrated previously [12].

## Results

### TMZ and Qct induce Cell Death

**Figure 1:**
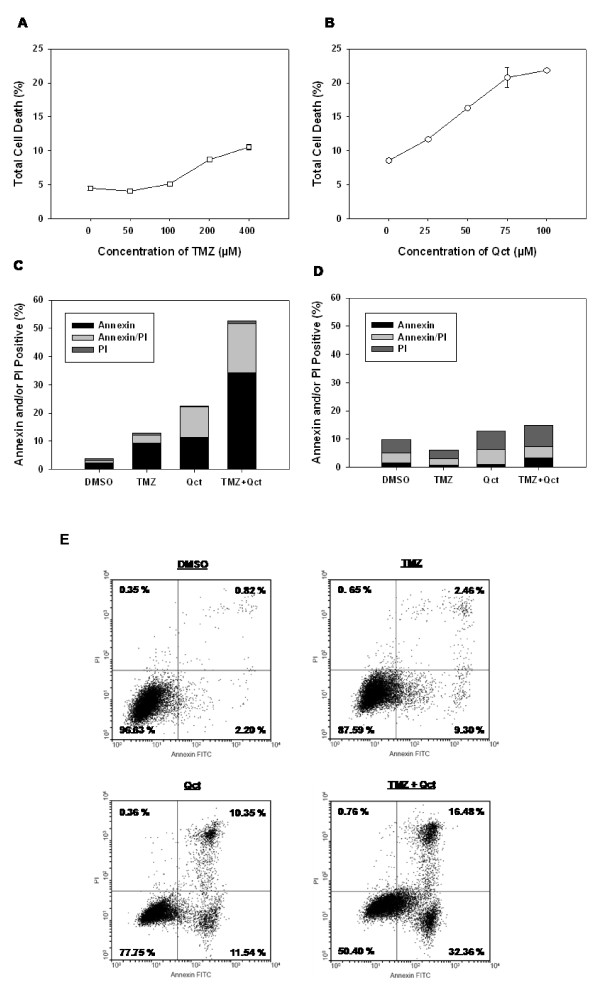
**The induction of apoptosis by TMZ, Qct or combination treatment**. DB-1 cell lines were subjected to varying dose of **A) **TMZ treatment (0-400 μM) for 48 hrs or **B) **Qct treatment for 24 hrs (0-100 μM). The cells were subjected to apoptotic analysis by Annexin/FITC staining by using BD FACS flow cytometer and the percentage of apoptosis was determined. **C) **The stacked percentages of apoptotic cells after TMZ treatment in DB-1 or **D) **SK Mel 28 cell lines Results are mean of duplicate experiments. **E) **Representative flow cytometric scatter plots of DB-1 cells. Early apoptotic cells can be visualized in the lower right quadrant, while late apoptotic/necrotic cells are shown in the upper right.

To establish a dose response to TMZ, DB-1 cells were treated with varying concentrations of TMZ (0-400 μM) for 48 hrs and the amount of cell death (apoptosis and necrosis) was determined by Annexin V-FITC/PI double staining and flow cytometry analysis. Consistent with a chemoresistant melanoma phenotype, DB-1 cells were resistant to TMZ-induced cell death at concentrations lower than 100 μM (Figure 1A). In contrast, Qct treatment (0-100 μM) resulted in dose dependent cell death at all concentrations studied (Figure 1B).

For subsequent combination studies, a concentration of 400 μM TMZ plus 75 μM Qct was used as those concentrations individually resulted in a distinguishable degree of apoptotic cell death (Figure 1C). To treat cells in combination TMZ was administered for 48 hrs followed by 24 hrs of Qct. In DB-1 cells treatment with TMZ or Qct induced 9.2% (± 0.4%) or 11.4% (± 0.4%) apoptosis (lower right quadrant of flow-cytometeric scatter plot, Figure 1E), respectively (Figure 1C). However, the combination treatment of TMZ plus Qct induced 34.25% (± 2.6%) apoptosis, which was greater than additive in these cell lines. Late apoptosis or necrosis (upper right quadrant of flow-cytometeric scatter plot, Figure 1E) was also increased in a more than additive manner. Apoptosis analysis was also carried out in p53 mutant SK Mel 28 cells and there was no significant increase in cell death across treatments (Figure 1D). Representative flow cytometeric scatter plots for DB-1 cells can be seen in Figure 1E.

### DNA Damage Response

**Figure 2:**
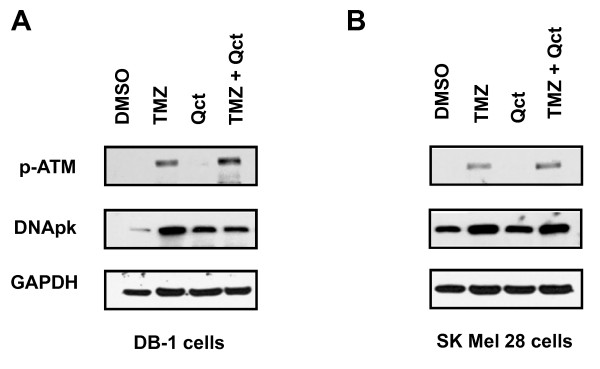
**The effect of TMZ, Qct or combination treatment on DNA Damage Response proteins**. Western blot analysis after treatment with vehicle (DMSO), TMZ 400 μM for 48 hrs followed by Qct 75 μM for 24 hrs. **A) **DB-1 or **B) **SK Mel 28 cell lines.

We next investigated the effect of Qct and TMZ on the DNA damage response. ATM is a protein kinase that is rapidly activated by DNA double strand breaks (DSBs) and is known to phosphorylate downstream target substrates such as p53. An increase in ATM phosphorylation at Ser 1981 (p-ATM) was observed in DB-1 cell lines treated with TMZ and a slight increase in p-ATM was observed by Qct treatment (Figure 2). The greatest increase in p-ATM was observed in the combination treatment. A similar increase in p-ATM was observed in SK Mel 28 cells, but Qct did not measurably activate this protein. Induction of DNApk, another DNA repair protein, was also observed in all the treatment groups (Figure 2).

**Figure 3:**
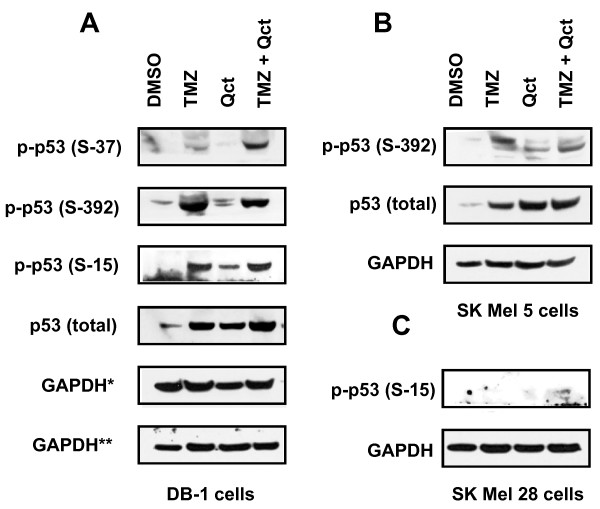
**The effect of TMZ, Qct or combination treatment on total p53 and phospho-p53**. Western blot analysis after treatment with vehicle (DMSO), TMZ 400 μM for 48 hrs followed by Qct 75 μM for 24 hrs. **A) **DB-1 or **B) **SK Mel 5 and **C) **SK Mel 28 cell lines. *indicates loading control for the different blots shown.

Because ATM was activated we investigated the effect of treatment on post-translational modification of p53 by evaluating phosphorylation of its serine moieties. In the DB-1 cells phosphorylation of p53 at serine 15, 37 and 392 was increased following treatment with TMZ or TMZ plus Qct (Figure 3A). The expression of total p53 also increased accordingly. We confirmed the sensitization effect in SK Mel 5, another p53 wild type cell line (Figure 3B). However, there was a minimal activation of p53 at serine 15 in SK Mel 28 cells (Figure 3C) and phosphorylation at other sites could not be detected (data not shown). No changes in the levels of total p53 were detected in the SK Mel 28 cells.

### Abrogation of apoptosis by ΔNp73

**Figure 4:**
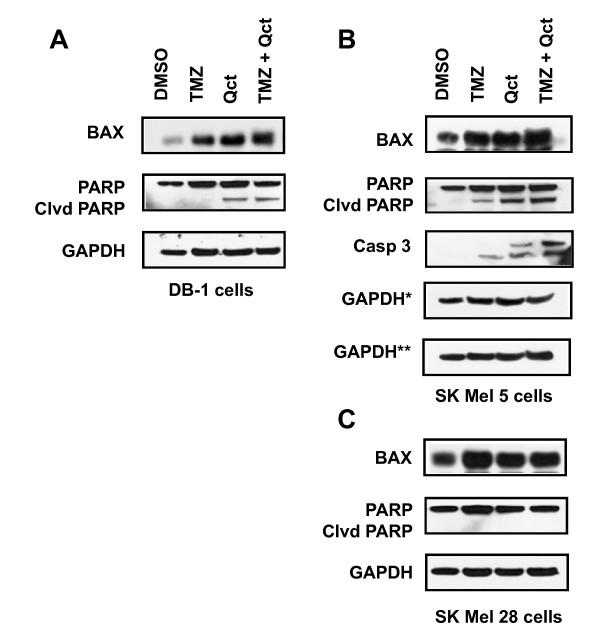
**The effect of TMZ, Qct or combination treatment on the levels of Bax and cleaved PARP**. Western blots of **A**) DB-1 **B) **SK Mel 5 and **C) **SK Mel 28 cells after treatment with vehicle (DMSO), TMZ 400 μM for 48 hrs followed by Qct 75 μM for 24 hrs. *indicates loading control for the different blots shown.

The effect of the treatments on the proteins downstream of p53, including Bax and cleaved PARP were also investigated (Figure 4). The levels of apoptosis (Figure 1C), cleaved PARP and caspase 3 (Figure 4A and 4B) did not correlate with the increased levels of p-ATM and phospho-p53 (Figures 2 and 3), indicating that signaling between p53 and its downstream targets was attenuated and thus blocked apoptosis. In the SK Mel 28 mutant cell lines, there were no changes in total p53 levels (data not shown) and levels of cleaved PARP (Figure 4C).

**Figure 5:**
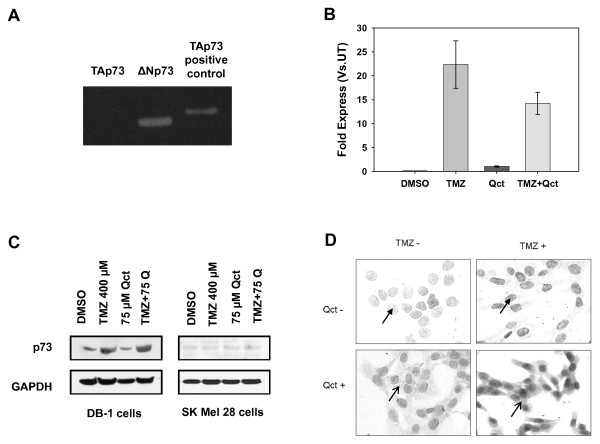
**Qct causes changes in p73 distribution**. **A) **Non-quantitative PCR in DB-1 cells for TAp73 (Lane 1), ΔNp73 (Lane 2) and TAp73 positive control using SK Mel 28 cells (Lane 3) following treatment with TMZ. TAp73 was undetectable in the DB-1 cell line. **B) **Real time RT-PCR for ΔNp73 in DB-1 cell lines treated with TMZ 400 μM for 48 hrs followed Qct 75 μM for 24 hrs. Results are Mean ± SEM of triplicate experiments in DB-1 cell lines. **C) **p73 expression by western blot analysis in DB-1 and SK Mel 28 cell lines treated with vehicle (DMSO), TMZ 400 μM for 48 hrs followed by Qct 75 μM for 24 hrs. **D) **Immunocytochemical analysis of p73 localization in DB-1 cell lines treated with vehicle (DMSO), TMZ 400 μM for 48 hrs followed by Qct 75 μM for 24 hrs. Solid arrowheads indicate nuclear staining in cells without Qct treatment, while line-type arrowheads indicate cytoplasmic staining following Qct treatment.

To investigate the abrogation of apoptosis in the cells treated with TMZ we examined the role of p53 family members, which have been shown to have inhibitory effects on p53 function. The p73 gene is a homologue of full length p53 and is involved in the transactivation of p53 target genes and thereby causes an induction in apoptosis and inhibition of cell proliferation [22]. Transcriptionally active or TAp73 is the active isoform of p73 frequently expressed in human tumors [23] and inhibited by either N terminally truncated p63 (ΔNp63) or ΔNp73. ΔNp73 can also antagonize p53. We performed RT-PCR for TAp73 and ΔNp73 to determine the isoforms expressed in DB-1 and SK-Mel28 cells. The only detectable isoform in DB-1 cells was ΔNp73 (Figure 5A) so DB-1 cells were used to further investigate the role of the p73 deletion mutant on apoptosis and chemoresistance. Following treatment with TMZ or the TMZ plus Qct combination there was an increase in the transcription of only ΔNp73 (Figure 5B). Western blot analysis also revealed a single band of increased protein levels of p73 in the DB-1 cells treated with TMZ alone or in combination with Qct and there was no change in protein levels of p73 in the SK Mel 28 cells (Figure 5C).

### Qct alters distribution of ΔNp73

The localization of p73 plays a major role in its activity so the effect of Qct on localization of p73 was investigated. The full length transcriptionally active form of p73 (TAp73) is normally found in the nucleus and can induce the transcription of downstream p53 target genes. However, when the ΔN form is in the nucleus, it can antagonize p53, which could be the reason why the levels of Bax (Figure 4) did not correlate with the increased levels of phospho-p53 (Figure 3) and the abrogation of apoptosis in the TMZ-treated cells. Consistent with previous reports of p73 localization, immunocytochemistry (ICC) analysis revealed the nuclear staining in the untreated cells that increased following treatment with TMZ (Figure 5D). In contrast, Qct treatment caused a re-distribution of ΔNp73 from the nucleus into cytoplasm as well as in the nucleus. The re-distribution was also observed with the combination treatment in DB-1 cell lines (Figure 5D) and indicates that the redistribution induced by Qct can reduce the antagonist effect of ΔNp73.

**Figure 6:**
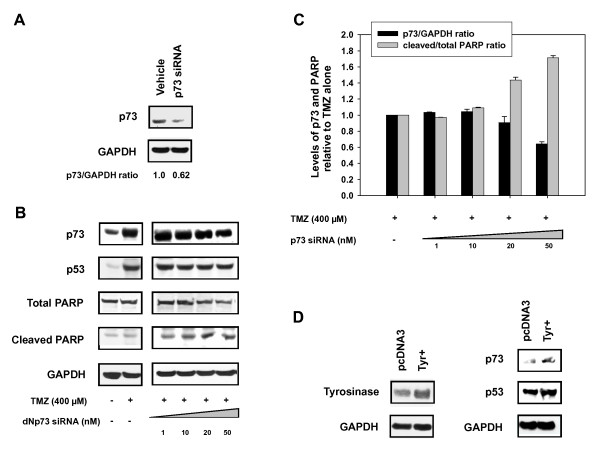
**Knockdown of p73 with siRNA restores PARP cleavage**. Western blot analysis after transfection with p73 siRNA in DB-1 cell lines **A) **at basal level and **B) **after TMZ treatment and **C) **Quantification of p73 to GAPDH and Cleaved PARP relative to uncleaved PARP. **D) **Transient transfection of tyrosinase DNA in DB-1 cell lines and western blot analysis of p53 and 73 expression.

To confirm the role of ΔNp73 in the attenuation of the apoptotic response siRNA experiments were performed. Naumann et al. [24] identified PARP cleavage as a hallmark of TMZ activity, so we investigated the effect of p73 siRNA on PARP levels. Treatment with TMZ resulted in no significant cleavage of PARP compared to control (Figure 6B). However, incubation with p73 siRNA in TMZ-treated cells knocked down the ΔNp73 protein levels and resulted in an increase in the ratio of cleaved to uncleaved PARP (Figure 6C). Taken together, these results confirmed the role of ΔNp73 as a p53 antagonist in the response to TMZ and suggests that Qct promotes apoptosis by inducing the translocation of ΔNp73 out of the nucleus.

### Melanogenesis and ΔNp73

Tyrosinase activity is increased as melanoma tumors develop and we have previously shown that tyrosinase activity is associated with an increase in p53 [11,12]. Because p53 can transcriptionally activate p73, we investigated if tyrosinase overexpression could induce the expression of p73. We transiently overexpressed tyrosinase (Figure 6D) and observed an increase in the expression of p53 and its transcriptional target p73 (Figure 6D). Therefore, tumorigenesis in melanoma, which coincides with the overexpression of tyrosinase, could also be associated with an increase in transcription of p53 antagonists and inducing resistance to chemotherapies. Targeting these antagonists through compounds such as quercetin could serve as an effective therapeutic or treatment modifier by restoring p53 activity.

## Discussion

### DNA Damage Response

We have shown for the first time that Qct can affect the cellular distribution of ΔNp73 and abrogate its anti-apoptotic effects. We also demonstrated in melanoma that Qct can affect the phosphorylation of p53, which is a key factor in mediating TMZ-induced apoptosis and a major determinant of cancer cell response to TMZ [25]. TMZ and DTIC methylate DNA at O^6 ^methyl guanine residues and cause mispairing with thymine and activation of MutSa-dependent mismatch repair resulting in apoptosis [26,27]. TMZ treatment during the present study caused an induction in total p53 and phosphorylation of its serine residues at 15, 37 and 392. Studies by Mhaidat NM et al. [28], illustrate that the sensitivity of melanoma cells to TMZ was dependent on their p53 status and associated G2/M arrest. We did confirm the central role of p53 with TMZ treatment and further demonstrated TMZ is a strong activator of ATM. ATM activation was enhanced by Qct, which provides a mechanism for the activation and stabilization of p53 while modulation of p73 likely contributes to increased p53 activity.

Even though apoptosis is considered the major mechanism of death by TMZ, [29] Qct also increased necrosis and indicates that apoptosis might not be the only therapeutic outcome when using combination treatments. Cell death reported here consists of an apoptotic fraction and a second fraction of cells that consisted of late apoptosis or necrosis. Necrosis as a mechanism of cell death can not be ruled out and is line with another study in malignant melanoma cells [30] that concluded TMZ-induced O^6^-methyl guanine triggers the apoptotic as well as necrotic pathway through the formation of DSBs. However, these pathways appear in part mediated by p53 as the SK Mel 28 cells were relatively void of treatment-induced cell death.

### Abrogation of apoptosis by ΔNp73

Another mechanism of action of Qct appears to be through the change in distribution of p73, allowing for an increase in p53 nuclear activity. p73 belongs to the p53 family of proteins that exhibit sequence homology [31] and they include p53, p63 and p73. Endogenous TAp73 is upregulated in response to DNA damage or treatment with chemotherapeutic drugs and provides antitumor activity, while the upregulation of ΔNp73 promotes resistance to these drugs [32]. The functionality of the p53 family members primarily depends on the nuclear localization of p73. Studies demonstrate that the export of the truncated form, ΔNp73, to the cytoplasm is a major inducer of p53 functionality [33]. In earlier studies with hepatocellular and cholangiocellular carcinomas, p73 was reported to be confined to the nucleus [34,35]. It was also found to be localized mainly in the nucleus of undifferentiating neuroblastomas [36].

Our ICC study demonstrates that expression of ΔNp73 was confined to the nucleus in control melanoma cells and this expression was even increased in the nucleus following TMZ treatment. Re-localization to the cytoplasm could indicate a shift in the balance of p53 to p73 in the nucleus and allow for transcription of p53 target genes, or may indicate a functional change in p73 itself. Inoue et al. [37] demonstrated that p73 undergoes active export which in part is mediated by nuclear export signal (NES) localized in the C terminus. Qct can modify phosphorylation which is required for a functional NES [38]. The change in localization could be therefore be related to changes in nuclear import and export signals and related changes in the overall distribution of p73.

The notion that p53 upregulates its own antagonist provides a unique mechanism for averting functional p53 which is commonly found in melanoma. We have previously demonstrated that tyrosinase overexpression could induce ROS which may induce p53 [11,12]. The upregulation of p53 in a tumor would not be beneficial unless it was transcribing an antagonistic protein such as ΔNp73. Box et al [14] have also proposed that p53 and antagonists are increased with melanogenesis. Our results with overexpression of tyrosinase are consistent with this notion. Abrogation of the protective effect of p53 antagonists would provide a valuable target for tumor therapy, especially since relatively non toxic molecules such as Qct can affect its translocation and activity.

## Conclusion

Our study is the first to demonstrate the effect of Qct on p73 distribution and suggests p53 antagonists are associated with treatment escape. Qct added to p53 wildtype melanoma cells abrogated chemoresistance and triggered a more than additive induction of apoptosis, which was associated with a cytosolic and nuclear localization of p73. We also propose that during melanogenesis p53 is induced in response to tyrosinase expression. The co-expression of p53 antagonists could block the apoptotic function of p53 and allow for continued development of tumors. Therefore modifiers of p53 antagonists, such as Qct, could serve as effective therapeutic or treatment modifiers. Alternatively, Qct be used to target antagonists for cancer prevention. The effect quercetin treatment on cells that express both the TA and ΔNp73 isoforms is unknown, but should be investigated.

## Abbreviations

ATM: Ataxia telangiectasia mutated; ALP: Alkaline phosphatase; Bax: Bcl-2-associated X protein; DMSO: Dimethyl sulphoxide; DNApk: DNA dependent protein kinase; DTIC: Dacarbazine; FITC: Fluorescein isothiocyanate; GAPDH: Glyceraldehyde 3 phosphate dehydrogenase; ICC: Immunohistochemistry; α-MEM: α Minimum essential medium; NQO1: Quinone oxidoreductase 1; PARP: Poly(ADP-Ribose) Polymerase; PI3KK: Phosphatidylinositol 3-kinase-like kinase; Qct: Quercetin; ROS: Reactive Oxygen Species; RT PCR: Real time polymerase chain reaction; TMZ: Temozolomide.

## Competing interests

The authors declare that they have no competing interests.

## Authors' contributions

TT and SS carried out molecular and cell biology experiments, data analysis, participated in the design of the studies and drafted the manuscript. GCM participated in molecular and cell biology experiments, data analysis, interpretation of results and written revisions. VRM contributed to the design of experiments and analysis of data. EEM participated in data analysis, interpretation of results and written revisions for the resubmission. KHL an RB conceived the studies and participated in design of the experiments. RB oversaw and coordinated the studies and finalized writing of the manuscript. All authors read and approved the final manuscript.

## Pre-publication history

The pre-publication history for this paper can be accessed here:

http://www.biomedcentral.com/1471-2407/10/282/prepub
